# Update: Influenza Activity — United States, October 2, 2016–February 4, 2017

**DOI:** 10.15585/mmwr.mm6606a2

**Published:** 2017-02-17

**Authors:** Lenee Blanton, Desiree Mustaquim, Noreen Alabi, Krista Kniss, Natalie Kramer, Alicia Budd, Shikha Garg, Charisse N. Cummings, Alicia M. Fry, Joseph Bresee, Wendy Sessions, Rebecca Garten, Xiyan Xu, Anwar Isa Abd Elal, Larisa Gubareva, John Barnes, David E. Wentworth, Erin Burns, Jacqueline Katz, Daniel Jernigan, Lynnette Brammer

**Affiliations:** 1 Influenza Division, National Center for Immunization and Respiratory Diseases, CDC.

This report summarizes U.S. influenza activity* during October 2, 2016–February 4, 2017,^†^ and updates the previous summary (*1*). Influenza activity in the United States began to increase in mid-December, remained elevated through February 4, 2017, and is expected to continue for several more weeks. To date, influenza A (H3N2) viruses have predominated overall, but influenza A (H1N1)pdm09 and influenza B viruses have also been identified.

## Virologic Surveillance

U.S. World Health Organization (WHO) and National Respiratory and Enteric Virus Surveillance System laboratories, which include both public health and clinical laboratories throughout the United States, contribute to virologic surveillance for influenza.

**FIGURE 1:**
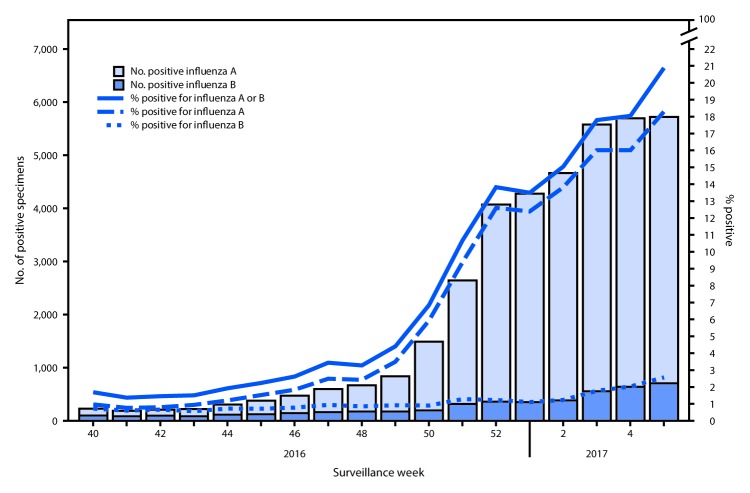
Number* and percentage of respiratory specimens testing positive for influenza reported by clinical laboratories, by influenza virus type and surveillance week — United States, October 2, 2016–February 4, 2017 * 38,244 (9.7%) of 392,907 tested were positive during October 2, 2016–February 4, 2017.

During October 2, 2016–February 4, 2017, clinical laboratories in the United States tested 392,901 respiratory specimens for influenza viruses, 38,244 (9.7%) of which were positive (Figure 1). During the week ending February 4, 2017 (week 5), 27,409 specimens were tested, 5,722 (20.9%) of which were positive for influenza. Among these, 5,017 (87.7%) were positive for influenza A viruses and 705 (12.3%) were positive for influenza B viruses.

**FIGURE 2:**
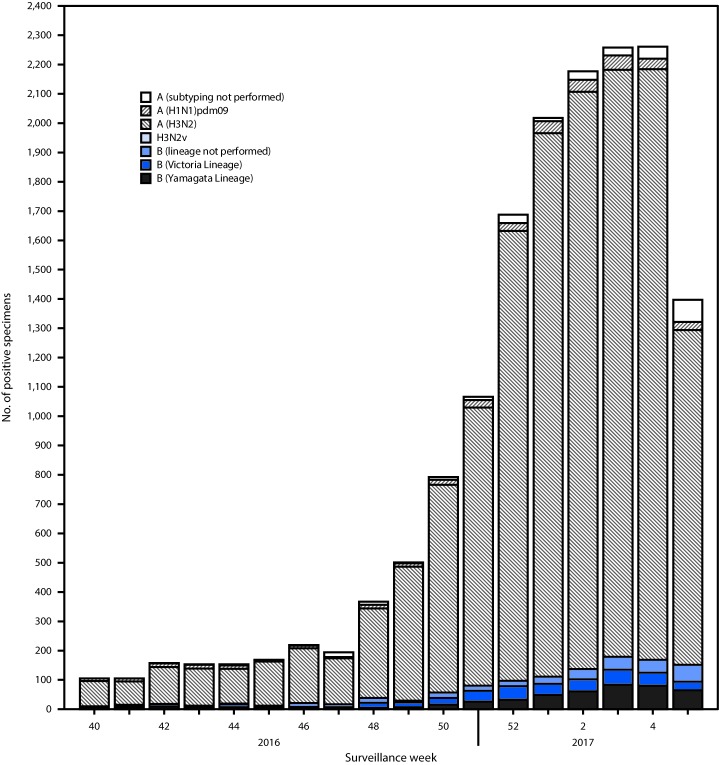
Number* of respiratory specimens testing positive for influenza reported by public health laboratories, by influenza virus type, subtype/lineage, and surveillance week — United States, October 2, 2016–February 4, 2017^†^ * N = 15,781. ^†^ As of February 10, 2017.

Public health laboratories in the United States tested 38,141 respiratory specimens collected during October 2, 2016–February 4, 2017. Among these, 15,781 were positive for influenza (Figure 2), 14,606 (92.6%) were positive for influenza A viruses, and 1,174 (7.4%) were positive for influenza B viruses. Among the 14,335 (98.1%) influenza A viruses subtyped, 13,973 (97.5%) were influenza A (H3N2) and 362 (2.5%) were influenza A (H1N1)pdm09 virus. Among the 851 (72.5%) influenza B viruses for which lineage was determined, 460 (54.1%) belonged to the B/Yamagata lineage and 391 (45.9%) belonged to the B/Victoria lineage.

Age was reported for 13,306 influenza-positive patients, among whom 1,048 (7.9%) were aged 0–4 years, 4,041 (30.4%) were aged 5–24 years, 4,029 (30.3%) were aged 25–64 years, and 4,188 (31.5%) were aged ≥65 years. Influenza A (H3N2) viruses predominated in each age group, representing a range of 82.3% of influenza-positives in persons aged 0–4 years to 93.6% in persons aged ≥65 years. The largest number of influenza B viruses were reported in persons aged 5–24 years.

## Novel Influenza A Viruses

Two human infections with a novel influenza A virus were reported during October 2, 2016–February 4, 2017. One patient from Iowa with exposure to swine in the week preceding illness was infected with an influenza A (H1N2) variant [(H1N2)v] virus.^§^ Another patient was infected with an avian lineage influenza A (H7N2) virus and reported close, prolonged unprotected exposure to the respiratory secretions of sick cats known to be infected with this virus at a New York City animal shelter. Neither patient was hospitalized; both recovered fully, and there was no evidence of human-to-human transmission in either instance.

## Antigenic and Genetic Characterization of Influenza Viruses

WHO collaborating laboratories in the United States are requested to submit a subset of influenza-positive respiratory specimens to CDC for further virus characterization. CDC characterizes influenza viruses through one or more laboratory tests, including genomic sequencing, antigenic characterization by hemagglutination inhibition (HI), and neutralization assays. Historically, HI data have been used most commonly to assess the similarity between vaccine viruses and circulating viruses to infer how well the vaccine might work until vaccine effectiveness estimates are available.^¶^ For all viruses characterized at CDC laboratories, next-generation sequencing is performed to determine the genetic identity of circulating viruses. The antigenic properties of viruses that cannot be characterized are inferred from viruses with matching genes whose antigenic profile is known.

CDC has genetically characterized 892 viruses (101 influenza A (H1N1)pdm09; 593 influenza A (H3N2); and 198 influenza B viruses) collected from October 1, 2016, through February 4, 2017. The hemagglutinin (HA) gene segment of all influenza A (H1N1)pdm09 viruses analyzed belonged to genetic group 6B.1. Influenza A (H3N2) virus HA gene segments analyzed belonged to genetic groups 3C.2a (567 viruses) or 3C.3a (26 viruses). Genetic group 3C.2a includes an emerging subgroup defined as 3C.2a1. The HA of influenza B/Victoria-lineage viruses all belonged to genetic group V1A. The HA of all influenza B/Yamagata-lineage viruses analyzed belonged to genetic group Y3.

During October 1, 2016–February 4, 2017, CDC antigenically characterized 484 influenza viruses (74 influenza A (H1N1)pdm09, 267 influenza A (H3N2), and 143 influenza B viruses). All 74 (100%) influenza A (H1N1)pdm09 viruses were antigenically similar to A/California/7/2009, the recommended influenza A (H1N1) component of the 2016–17 Northern Hemisphere vaccine. Among 267 influenza A (H3N2) viruses, 258 (96.6%) were antigenically similar to the A/Hong Kong/4801/2014–like cell propagated reference virus belonging to genetic group 3C.2a, which is the recommended influenza A (H3N2) component of the 2016–17 Northern Hemisphere vaccine. Seventy (90.9%) of 77 influenza B/Victoria-lineage viruses were antigenically similar to B/Brisbane/60/2008, which is the recommended influenza B component of the 2016–17 Northern Hemisphere trivalent and quadrivalent vaccines. All 66 (100%) influenza B/Yamagata-lineage viruses were antigenically similar to B/Phuket/3073/2013, the recommended influenza B component of the 2016–17 Northern Hemisphere quadrivalent vaccine.

## Antiviral Resistance of Influenza Viruses

The WHO Collaborating Center for Surveillance, Epidemiology, and Control of Influenza at CDC tested 807 influenza virus specimens (94 influenza A (H1N1)pdm09, 519 influenza A (H3N2), and 194 influenza B viruses) collected in the United States from October 1, 2016, through February 4, 2017, for resistance to the influenza neuraminidase inhibitor antiviral medications oseltamivir, zanamivir, and peramivir, drugs currently approved for use against seasonal influenza. All 807 influenza viruses tested were found to be sensitive to all three antiviral medications. An additional 114 influenza A (H3N2) viruses were tested for resistance to oseltamivir and zanamivir, and were found to be sensitive to both antiviral medications.

## Outpatient Illness Surveillance

**FIGURE 3:**
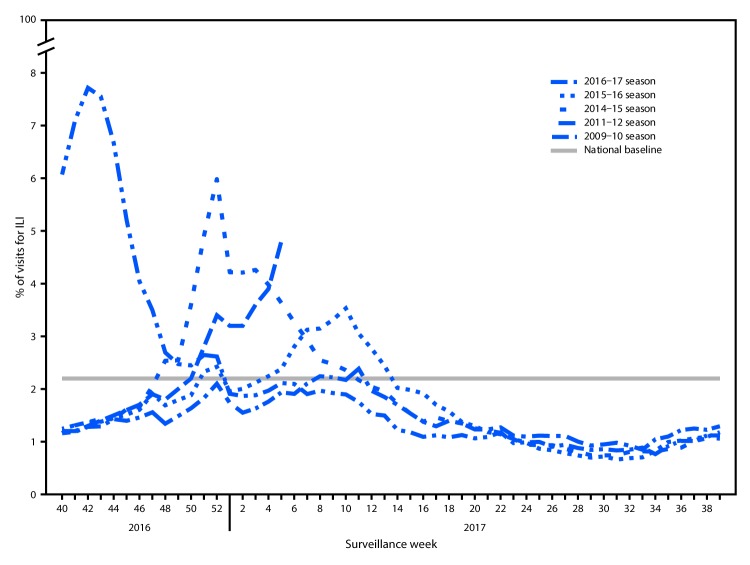
Percentage of visits for influenza-like illness (ILI)* reported to CDC, by surveillance week — Outpatient Influenza-Like Illness Surveillance Network, United States, 2016–17 influenza season and selected previous influenza seasons * Defined as fever (≥100°F [≥37.8°C]), oral or equivalent, and cough and/or sore throat, without a known cause other than influenza.

From October 2, 2016, through February 4, 2017, the weekly percentage of outpatient visits for influenza-like illness (ILI)** reported by approximately 2,000 U.S. Outpatient ILI Surveillance Network (ILINet) providers in 50 states, New York City, Chicago, the U.S. Virgin Islands, Puerto Rico, and the District of Columbia, has ranged from 1.2% to 4.8%. The percentage exceeded the national baseline^††^ of 2.2% for 8 consecutive weeks, from the weeks ending December 17, 2016–February 4, 2017 (weeks 50–5) (Figure 3). During the previous five influenza seasons, the peak weekly percentages of outpatient visits for ILI ranged from 2.4%–6.1% and remained above baseline levels for an average of 13 weeks (range = 1–20 weeks). For the week ending February 4, 2017 (week 5), the percentage of outpatient visits for ILI was 4.8%, and all 10 U.S. Department of Health and Human Services (HHS) regions^§§^ reported ILI activity at or above region-specific baseline levels.

Data collected in ILINet are used to produce a measure of ILI activity^¶¶^ by jurisdiction. During the week ending February 4, 2017, New York City and 23 states (Alabama, Arkansas, Connecticut, Georgia, Hawaii, Indiana, Kansas, Louisiana, Minnesota, Mississippi, Missouri, New Jersey, New Mexico, New York, North Carolina, Oklahoma, Pennsylvania, South Carolina, South Dakota, Tennessee, Texas, Virginia, and Wyoming) experienced high ILI activity; 10 states (California, Colorado, Florida, Illinois, Iowa, Michigan, Nebraska, North Dakota, Oregon, and Wisconsin) experienced moderate ILI activity; Puerto Rico and eight states (Alaska, Arizona, Kentucky, Maryland, Massachusetts, Nevada, Rhode Island, and West Virginia) experienced low ILI activity; nine states (Delaware, Idaho, Maine, Montana, New Hampshire, Ohio, Utah, Vermont, and Washington) experienced minimal ILI activity; and the District of Columbia had insufficient data to calculate an ILI activity level.

## Geographic Spread of Influenza Activity

Influenza activity levels reported by state and territorial epidemiologists indicate the geographic spread of influenza viruses. For the week ending February 4, 2017 (week 5), Puerto Rico and 43 states (Alabama, Alaska, Arkansas, California, Connecticut, Delaware, Florida, Georgia, Idaho, Illinois, Iowa, Kansas, Kentucky, Louisiana, Maine, Maryland, Massachusetts, Michigan, Minnesota, Mississippi, Missouri, Montana, Nebraska, Nevada, New Hampshire, New Jersey, New Mexico, New York, North Carolina, North Dakota, Ohio, Oklahoma, Oregon, Pennsylvania, Rhode Island, South Carolina, South Dakota, Texas, Vermont, Virginia, Washington, Wisconsin, and Wyoming) reported widespread activity.*** Guam and six states (Arizona, Colorado, Indiana, Tennessee, Utah, and West Virginia) reported regional activity. The District of Columbia and one state (Hawaii) reported local activity, and the U.S. Virgin Islands reported no influenza activity. During the previous five influenza seasons, the peak number of jurisdictions reporting widespread activity in a single week during each season has ranged from 20 in the 2011–12 season to 48 during the 2012–13 season.

## Influenza-Associated Hospitalizations

CDC monitors hospitalizations associated with laboratory-confirmed influenza infection in adults and children through the Influenza Hospitalization Surveillance Network (FluSurv-NET),^†††^ which covers approximately 27 million persons (9% of the U.S. population). From October 1, 2016, through February 4, 2017, 6,804 laboratory-confirmed influenza-associated hospitalizations were reported, with a cumulative incidence for all age groups of 24.3 per 100,000 population. Persons aged ≥65 years had the highest rate of laboratory-confirmed influenza-associated hospitalization and accounted for approximately 60% of reported influenza-associated hospitalizations.

The cumulative hospitalization rate (per 100,000 population) during October 1, 2016–February 4, 2017 was 13.6 among children aged 0–4 years, 4.8 among children and adolescents aged 5–17 years, 7.3 among adults aged 18–49 years, 23.5 among adults aged 50–64 years, and 113.4 among adults aged ≥65 years. Among all hospitalizations reported during October 1, 2016–February 4, 2017, a total of 6,367 (93.6%) were associated with influenza A virus, 395 (5.8%) with influenza B virus, 21 (0.3%) with influenza A and B virus coinfection, and 21 (0.3%) with influenza virus for which the type was not determined. Among 1,729 patients with influenza A subtype information, 1,701 (98.4%) were infected with influenza A (H3N2) virus and 28 (1.6%) were infected with influenza A (H1N1)pdm09 virus.

Complete medical chart abstraction data were available for 796 (11.7%) hospitalized patients with laboratory-confirmed influenza as of February 4, 2017. Among 744 hospitalized adults with complete medical chart abstraction, 703 (94.5%) had at least one underlying medical condition that placed them at high risk for influenza-associated complications. The most commonly reported medical conditions were cardiovascular disease (47.1%), metabolic disorders (39.7%), and obesity (38.3%). Among 52 hospitalized children with complete medical chart abstraction, 28 (53.8%) had at least one underlying medical condition, the most commonly reported being asthma (15.4%), chronic lung disease (15.4%), and neurologic disorder (15.4%). Among 59 hospitalized women of childbearing age (15–44 years), 20 (33.9%) were pregnant.

## Pneumonia and Influenza-Attributed Mortality

CDC tracks pneumonia and influenza (P&I)-attributed deaths through the National Center for Health Statistics (NCHS) Mortality Reporting System. The percentages of deaths attributed to P&I are released 2 weeks after the week of death to allow for collection of sufficient data to produce a stable P&I mortality percentage. Weekly mortality surveillance data includes a combination of machine coded and manually coded causes of death collected from death certificates. There is a backlog of data requiring manual coding within the NCHS mortality surveillance data. The percentages of deaths attributable to P&I are higher among manually coded records than the more rapidly available machine coded records and might result in initially reported P&I percentages that are lower than percentages calculated from final data. Initiatives continue to reduce and monitor the number of records awaiting manual coding.

Based on data from NCHS available on February 9, 2017, 7.9% (2,691 of 33,868) of all U.S. deaths occurring during the week ending January 21, 2017 (week 3) were attributed to P&I. This percentage is above the epidemic threshold^§§§^ of 7.4% for week 3. Since October 2, 2016 the weekly percentage of deaths attributed to P&I has ranged from 5.6% to 7.9% and has exceeded the epidemic threshold for three consecutive weeks, from the weeks ending January 7–21 (weeks 1–3), this season. During the previous five influenza seasons, the peak weekly percentage of deaths attributable to P&I ranged from 8.2% in the 2015–16 season to 11.1% in the 2012–13 season.

## Influenza-Associated Pediatric Mortality

As of February 4, 2017 (week 5), 20 laboratory-confirmed influenza-associated pediatric deaths that occurred during the 2016–17 season were reported to CDC. Of the 20 deaths, nine were associated with an influenza A (H3N2) virus infection, one was associated with an influenza A (H1N1)pdm09 virus infection, five were associated with an influenza A virus infection for which no subtyping was performed, four were associated with an influenza B virus infection, and one was associated with an influenza virus for which the type was not determined. Since influenza-associated pediatric mortality became a nationally notifiable condition in 2004, the total number of influenza-associated pediatric deaths per season has ranged from 37 to 171; excluding the 2009 pandemic, when 358 pediatric deaths were reported to CDC from April 15, 2009, through October 2, 2010.

## Discussion

Influenza activity in the United States began to increase in mid-December and remained elevated as of February 4, 2017. During the most recent weeks, decreases in activity have been observed in the Northwest (HHS Region 10), while activity has continued to increase in the remainder of the country. During October 2, 2016–February 4, 2017, influenza A (H3N2) viruses accounted for the majority of circulating influenza viruses, but influenza A (H1N1)pdm09 and influenza B viruses also were identified. Influenza activity has been moderate so far this season, and severity indicators are within the range of what has been observed during previous seasons when influenza A (H3N2) viruses predominated. Elevated influenza activity in parts of the United States is expected for several more weeks.

Interim estimates of vaccine effectiveness based on data collected from November 28, 2016, through February 4, 2017, indicate that overall the influenza vaccine has been 48% (95% confidence interval [CI] = 37%–57%) effective in preventing influenza-related medical visits across all age groups, and specifically was 43% (CI = 29%–54%) and 73% (CI = 54%–84%) effective in preventing medical visits associated with influenza A (H3N2) and influenza B, respectively (*2*). Annual influenza vaccination is the first and best defense to protect against influenza infection. Depending on the vaccine formulation (trivalent or quadrivalent), influenza vaccines can protect against three or four different influenza viruses. Even during seasons when vaccine effectiveness is reduced, vaccination can offer substantial benefit and might reduce the likelihood of severe outcomes such as hospitalization and death.

Although health care providers should continue to offer and encourage vaccination to all unvaccinated persons aged ≥6 months as long as influenza viruses are circulating, influenza antiviral medications are an important adjunct to vaccination in the treatment and prevention of influenza. No antiviral resistance to oseltamivir, zanamivir, or peramivir has been identified among influenza viruses collected since October 1, 2016. Treatment as soon as possible with influenza antiviral medications is recommended for patients with confirmed or suspected influenza who have severe, complicated, or progressive illness; who require hospitalization; or who are at high risk for influenza complications.^¶¶¶^ Antiviral treatment should not be withheld from high-risk or severely ill patients with suspected influenza infection, even if rapid antigen-detection influenza diagnostic test results are negative (*3*). Generic oseltamivir was approved by the Food and Drug Administration on August 3, 2016 (*4*), and became available in December 2016.

An unusual outbreak of avian lineage influenza A (H7N2) virus infection among cats in an animal shelter in New York City was first reported to public health officials on December 14, 2016. Approximately 350 persons with exposure to infected cats during this outbreak were screened or tested for infection and only one human infection with avian influenza A (H7N2) was identified (*5*). This is the first influenza A (H7N2) virus infection in humans identified in the United States since 2003 and the first known human infection with an influenza A virus likely acquired through exposure to an ill cat. The finding of an avian lineage influenza virus in an unexpected host, such as a domestic cat, or any human infection with a nonhuman influenza virus is concerning. Early identification and investigation of human infections with novel influenza A viruses are critical so that the risk of infection can be more fully understood and appropriate public health measures can be taken. If clinical laboratories test a respiratory specimen that they cannot type or subtype using commercially available rapid or molecular influenza diagnostic tests, they should contact their state public health laboratory to facilitate transport of specimens for additional testing. Public health laboratories should immediately send virus specimens that they cannot type or subtype using standard methods to CDC and submit all specimens that are otherwise unusual as soon as possible after identification.

Influenza surveillance reports for the United States are posted online weekly (https://www.cdc.gov/flu/weekly). Additional information regarding influenza viruses, influenza surveillance, influenza vaccine, influenza antiviral medications, and novel influenza A infections in humans is online (https://www.cdc.gov/flu).

**Summary**

**What is already known about this topic?**

CDC collects, compiles, and analyzes data on influenza activity year round in the United States. Timing of influenza activity and predominant circulating influenza viruses vary by season.

**What is added by this report?**

Influenza activity in the United States began to increase in mid-December, remained elevated through February 4, 2016, and is expected to continue for several more weeks. During October 2, 2016–February 4, 2017, influenza A (H3N2) viruses were identified most frequently, but influenza A (H1N1)pdm09 and influenza B viruses were also reported. No antiviral resistance to oseltamivir, zanamivir, or peramivir has been identified among influenza viruses tested to date.

**What are the implications for public health practice?**

Elevated influenza activity in parts of the United States is expected for several more weeks. Influenza vaccination remains the most effective way to prevent influenza illness. Antiviral medications are an important adjunct to vaccination in the treatment and prevention of influenza. Early treatment with neuraminidase inhibitor antiviral medications is recommended for patients with severe, complicated, or progressive influenza illness and those at higher risk for influenza complications, including adults aged ≥65 years.

## Footnotes

* The CDC influenza surveillance system collects five categories of information from eight data sources: 1) viral surveillance (U.S. World Health Organization collaborating laboratories, the National Respiratory and Enteric Virus Surveillance System, and novel influenza A virus case reporting); 2) outpatient illness surveillance (U.S. Outpatient Influenza-Like Illness Surveillance Network); 3) mortality (the National Center for Health Statistics Mortality Surveillance System and influenza-associated pediatric mortality reports); 4) hospitalizations (FluSurv-NET, which includes the Emerging Infections Program and surveillance in three additional states); and 5) summary of the geographic spread of influenza (state and territorial epidemiologist reports). https://www.cdc.gov/flu/weekly/fluactivitysurv.htm.

† Data as of February 10, 2017.

§ Influenza viruses that circulate in swine are called swine influenza viruses when isolated from swine, but are called variant influenza viruses when isolated from humans. Seasonal influenza viruses that circulate worldwide in the human population have important antigenic and genetic differences from influenza viruses circulating in swine.

¶ A virus is considered “reference virus-like” if its hemagglutination inhibition (HI) or neutralization focus reduction assay (FRA) titer is within fourfold of the homologous HI/FRA titer of the reference strain. A virus is considered as low to the reference virus if there is an eightfold or greater reduction in the HI or FRA titer when compared with the homologous HI or FRA titer of the reference strain.

** Defined as a fever (temperature ≥100°F [≥37.8°C]), oral or equivalent, and cough and/or sore throat, without a known cause other than influenza.

†† The national and regional baselines are the mean percentage of visits for influenza-like illness (ILI) during noninfluenza weeks for the previous three seasons plus two standard deviations. Noninfluenza weeks are defined as periods of ≥2 consecutive weeks in which each week accounted for <2% of the season’s total number of specimens that tested positive for influenza. National and regional percentages of patient visits for ILI are weighted based on state population. Use of the national baseline for regional data is not appropriate.

§§ The 10 regions include the following jurisdictions: Region 1: Connecticut, Maine, Massachusetts, New Hampshire, Rhode Island, and Vermont; Region 2: New Jersey, New York, Puerto Rico, and the U.S. Virgin Islands; Region 3: Delaware, District of Columbia, Maryland, Pennsylvania, Virginia, and West Virginia; Region 4: Alabama, Florida, Georgia, Kentucky, Mississippi, North Carolina, South Carolina, and Tennessee; Region 5: Illinois, Indiana, Michigan, Minnesota, Ohio, and Wisconsin; Region 6: Arkansas, Louisiana, New Mexico, Oklahoma, and Texas; Region 7: Iowa, Kansas, Missouri, and Nebraska; Region 8: Colorado, Montana, North Dakota, South Dakota, Utah, and Wyoming; Region 9: Arizona, California, Hawaii, Nevada, American Samoa, Commonwealth of the Northern Mariana Islands, Federated States of Micronesia, Guam, Marshall Islands, and Republic of Palau; Region 10: Alaska, Idaho, Oregon, and Washington.

¶¶ Activity levels are based on the percentage of outpatient visits in a jurisdiction attributed to ILI and are compared with the average percentage of ILI visits that occur during weeks with little or no influenza virus circulation. Activity levels range from minimal, corresponding to ILI activity from outpatient clinics at or below the average, to high, corresponding to ILI activity from outpatient clinics much higher than the average. Because the clinical definition of ILI is nonspecific, not all ILI is caused by influenza; however, when combined with laboratory data, the information on ILI activity provides a clearer picture of influenza activity in the United States.

*** Levels of activity are 1) no activity; 2) sporadic: isolated laboratory-confirmed influenza cases or a laboratory-confirmed outbreak in one institution, with no increase in activity; 3) local: increased ILI, or two or more institutional outbreaks (ILI or laboratory-confirmed influenza) in one region of the state, with recent laboratory evidence of influenza in that region; virus activity no greater than sporadic in other regions; 4) regional: increased ILI activity or institutional outbreaks (ILI or laboratory-confirmed influenza) in two or more outbreaks, but less than half of the regions in the state with recent laboratory evidence of influenza in those regions; and 5) widespread: increased ILI activity or institutional outbreaks (ILI or laboratory-confirmed influenza) in at least half the regions in the state, with recent laboratory evidence of influenza in the state.

††† FluSurv-NET conducts population-based surveillance for laboratory-confirmed, influenza-associated hospitalizations in children and adolescents aged <18 years (since the 2003–04 influenza season) and adults aged ≥18 years (since the 2005–06 influenza season). The FluSurv-NET covers approximately 70 counties in the 10 Emerging Infections Program states (California, Colorado, Connecticut, Georgia, Maryland, Minnesota, New Mexico, New York, Oregon, and Tennessee) and additional Influenza Hospitalization Surveillance Project (IHSP) states. IHSP began during the 2009–10 season to enhance surveillance during the 2009 H1N1 pandemic. IHSP sites included Iowa, Idaho, Michigan, Oklahoma, and South Dakota during the 2009–10 season; Idaho, Michigan, Ohio, Oklahoma, Rhode Island, and Utah during the 2010–11 season; Michigan, Ohio, Rhode Island, and Utah during the 2011–12 season; Iowa, Michigan, Ohio, Rhode Island, and Utah during the 2012–13 season; and Michigan, Ohio, and Utah during the 2013–14, 2014–15, 2015–16, and 2016–17 seasons. Cumulative unadjusted incidence rates are calculated using CDC’s National Center for Health Statistics population estimates for the counties included in the surveillance catchment area. Laboratory confirmation is dependent on clinician-ordered influenza testing, and testing for influenza often is underutilized because of the poor reliability of rapid test results and greater reliance on clinical diagnosis for influenza. Therefore, cases identified as part of influenza hospitalization surveillance likely are an underestimation of the actual number of persons hospitalized with influenza.

§§§ The seasonal baseline proportion of pneumonia and influenza (P&I) deaths is projected using a robust regression procedure, in which a periodic regression model is applied to the observed percentage of deaths from P&I that were reported by the National Center for Health Statistics Mortality Surveillance System during the preceding 5 years. The epidemic threshold is set at 1.645 standard deviations above the seasonal baseline.

¶¶¶ Persons at higher risk include 1) children aged <2 years; 2) adults aged ≥65 years; 3) persons with chronic pulmonary conditions (including asthma), cardiovascular disease (except hypertension alone), renal, hepatic, hematologic (including sickle cell) disease, metabolic disorders (including diabetes mellitus), or neurologic and neurodevelopmental conditions (including disorders of the brain, spinal cord, peripheral nerves, and muscles, such as cerebral palsy, epilepsy [seizure disorders], stroke, intellectual disability [mental retardation], moderate to severe developmental delay, muscular dystrophy, or spinal cord injury); 4) persons with immunosuppression, including that caused by medications or by human immunodeficiency virus infection; 5) women who are pregnant or postpartum (within 2 weeks after delivery); 6) persons aged ≤18 years who are receiving long-term aspirin therapy; 7) American Indians/Alaska Natives; 8) persons with extreme obesity (i.e., body mass index ≥40); and 9) residents of nursing homes and other chronic care facilities.
